
JOURNAL INFORMATION
==============================
NLM Title Abbreviation: Medizinrecht
Journal ID: Medizinrecht
NLM Journal ID: 8309354

Medizinrecht

ISSN: 0723-8886
EISSN: 1433-8629

ARTICLE INFORMATION
==============================
PMCID: PMC10013277
PMID: 36937331
DOI: 10.1007/s00350-023-6419-7
Article ID: 6419
Article version: 1
Subjects: Aufsätze

Das Gesetz zum Schutz von Menschen mit Behinderung in der Triage

Grundrechtsschutz in pandemischen Ausnahmesituationen

Eufinger Alexander
Klemm Victoria
grid.449475.f 0000 0001 0669 6924 Wiesbaden Business School, Hochschule RheinMain, Postfach 3251, 65022 Wiesbaden, Deutschland
Electronic publication date: 2023 Mar 14
Print publication date: 2023
Volume: 41
Issue: 3
First page: 200
Last page: 204
Copyright: © Der/die Autor(en) 2023
License: Open Access Dieser Artikel wird unter der Creative Commons Namensnennung 4.0 International Lizenz veröffentlicht, welche die Nutzung, Vervielfältigung, Bearbeitung, Verbreitung und Wiedergabe in jeglichem Medium und Format erlaubt, sofern Sie den/die ursprünglichen Autor(en) und die Quelle ordnungsgemäß nennen, einen Link zur Creative Commons Lizenz beifügen und angeben, ob Änderungen vorgenommen wurden.
Die in diesem Artikel enthaltenen Bilder und sonstiges Drittmaterial unterliegen ebenfalls der genannten Creative Commons Lizenz, sofern sich aus der Abbildungslegende nichts anderes ergibt. Sofern das betreffende Material nicht unter der genannten Creative Commons Lizenz steht und die betreffende Handlung nicht nach gesetzlichen Vorschriften erlaubt ist, ist für die oben aufgeführten Weiterverwendungen des Materials die Einwilligung des jeweiligen Rechteinhabers einzuholen.
Weitere Details zur Lizenz entnehmen Sie bitte der Lizenzinformation auf http://creativecommons.org/licenses/by/4.0/deed.de.
License URL: https://creativecommons.org/licenses/by/4.0/

issue-copyright-statement: © C.H. Beck Verlag oHG, München und Springer-Verlag GmbH Deutschland, ein Teil von Springer Nature 2023

==============================

### Grundrechtsschutz in pandemischen Ausnahmesituationen

**Alexander Eufinger und Victoria Klemm**

#### A. Einführung

Im Zuge der Corona-Pandemie ist das französische Wort *Triage* (von französisch *„trier"* – sortieren) zum Schreckgespenst im Gesundheitswesen geworden[1]. So wurde befürchtet, dass die intensivmedizinischen Kapazitäten für die Anzahl der zu behandelnden Patienten – insbesondere von beatmungspflichtigen COVID-19-Patienten – nicht ausreichen würden. Genährt wurde diese Angst auch durch die schrecklichen Bilder aus der norditalienischen Stadt Bergamo. In der deutschen Gesundheitspolitik galt daher die apodiktische Aussage: *„Keine Zustände wie in Bergamo zu bekommen"*. Nachvollziehbarerweise waren entsprechende Befürchtungen vor den Folgen einer Triage bei Menschen mit Behinderungen besonders stark ausgeprägt. Dies führte zu einer Verfassungsbeschwerde von acht Menschen mit Behinderungen, welche am 16.12.2021 vom BVerfG entschieden wurde. Kernaussage des Beschlusses war, dass sich aus Art. 3 Abs. 3 S. 2 GG für den Staat der Auftrag ergebe, Menschen wirksam vor einer Benachteiligung wegen ihrer Behinderung durch Dritte zu schützen. Daher sei der Gesetzgeber gefordert, *„unverzüglich geeignete Vorkehrungen [zu] treffen"*, um Menschen mit Behinderung vor einer Diskriminierung bei der pandemiebedingten Triage zu schützen[2]. Bestehe das Risiko, dass Menschen in einer Triage-Situation bei der Zuteilung intensivmedizinischer Behandlungsressourcen wegen einer Behinderung benachteiligt werden, verdichte sich der Schutzauftrag aus Art. 3 Abs. 3 S. 2 GG zu einer konkreten Pflicht des Staates, hiergegen wirksame Vorkehrungen zu treffen. Vorliegend hatte das Verfassungsgericht für die Beschwerdeführenden das konkrete Risiko gesehen, wegen ihrer Behinderung bei der Zuteilung knapper, überlebenswichtiger intensivmedizinischer Ressourcen benachteiligt zu werden.

Um dem Beschluss des BVerfG nachzukommen und damit die bestehende staatliche Schutzpflicht aus Art. 3 Abs. 3 S. 2 GG zu erfüllen, hat der Bundestag am 10.11.2022 den Entwurf eines Zweiten Gesetzes zur Änderung des Infektionsschutzgesetzes verabschiedet, mit dem die Triage in einer besonderen Ausnahmesituation geregelt wird[3]. Das Triage-Gesetz beansprucht, für die Zuteilungsentscheidung maßgebliche Kriterien und Verfahrensvorschriften zu regeln. Der folgende Beitrag stellt die Inhalte des Triage-Gesetzes vor und würdigt diese – insbesondere unter Berücksichtigung der Entscheidung des BVerfG.

#### B. Der Entwurf des § 5c IfSG und Würdigung

Wie eingangs erwähnt, soll das Triage-Gesetz den vom BVerfG festgestellten Mangel, dass Menschen mit Behinderung bei der pandemiebedingten Triage einem Diskriminierungsrisiko unterlägen, heilen. Gesetzgeberische Aufgabe ist es hierbei, durch eine gesetzliche Regelung hinreichend wirksamen Schutz vor Benachteiligung zu verwirklichen. Im Folgenden werden die Neuerungen zur besseren Einordnung mit der bis dato Anwendung findenden S1-Leitlinie der Deutschen interdisziplinären Vereinigung für Intensiv- und Notfallmedizin (DIVI) *„Entscheidungen über die Zuteilung intensivmedizinischer Ressourcen im Kontext der COVID-19-Pandemie"* in der dritten überarbeiteten Fassung vom 14.12.2021 verglichen.

##### *I. § 5c Abs. 1 IfSG-E*

1. Inhalt

Das Triage-Gesetz sieht eine Änderung des Infektionsschutzgesetzes, genauer die Ergänzung von § 5c IfSG, vor. In § 5c IfSG-E soll die Zuteilung von intensivmedizinischen Behandlungskapazitäten bei pandemiebedingt aufgetretenem Ressourcenmangel geregelt werden. Zunächst stellt § 5c Abs. 1 IfSG-E klar, dass niemand bei einer ärztlichen Entscheidung über die Zuteilung von pandemiebedingt nicht ausreichend vorhandenen überlebenswichtigen intensivmedizinischen Behandlungskapazitäten (Zuteilungsentscheidung) insbesondere wegen einer Behinderung, der Gebrechlichkeit, des Alters, der ethnischen Herkunft[4], der Religion oder Weltanschauung, des Geschlechts oder der sexuellen Orientierung benachteiligt werden darf. § 5c Abs. 1 IfSG-E wiederholt damit in modifizierter Weise Art. 3 Abs. 3 S. 2 GG sowie § 1 AGG. Allerdings wird im Unterschied zum Grundgesetz auch die Ungleichbehandlung aufgrund des *„Alters"* sowie der *„Gebrechlichkeit"* mitaufgenommen. Das pönalisierte Merkmal der Gebrechlichkeit geht zudem über § 1 AGG hinaus[5]. Anders als das AGG knüpft § 5c Abs. 1 IfSG-E jedoch nicht an arbeits- oder zivilrechtliche Schuldverhältnisse an, sondern gilt unabhängig von den vertraglichen Beziehungen zwischen Patientin oder Patient und Behandelnden. Dennoch kann nach dem Willen des Gesetzgebers für die Auslegung der übrigen Kriterien auf die zu den Diskriminierungsmerkmalen des AGG ergangenen Entscheidungen zurückgegriffen werden[6].

Prof. Dr. iur. Alexander Eufinger,
Wiesbaden Business School, Hochschule RheinMain,
Postfach 3251, 65022 Wiesbaden, Deutschland

Victoria Klemm, Wissenschaftliche Mitarbeiterin,
Wiesbaden Business School, Hochschule RheinMain,
Postfach 3251, 65022 Wiesbaden, Deutschland

1) Vgl. hierzu auch *Brettel/Schuhr*, MedR 2022, 801, 802.
2) Vgl. BVerfG, Beschl. v. 16.12.2021 – 1 BvR 1541/20 –, MedR 2022, 212.
3) Gesetzentwurf der BReg., Entwurf eines Zweiten Gesetzes zur Änderung des Infektionsschutzgesetzes; abrufbar unter: https://dserver.bundestag.de/btd/20/038/2003877.pdf; zuletzt aufgerufen am 11.2.2023.
4) Verzichtet wurde auf die Aufnahme des Kriteriums der „Rasse". Die Europäische Union weist in Erwägungsgrund 6 der Richtlinie 2000/43/EG des Rates vom 29.6.2000 zur Anwendung des Gleichheitsgrundsatzes ohne Unterschied der Rasse oder der ethnischen Herkunft Theorien über die Existenz unterschiedlicher Rassen zurück. Ungeachtet dessen ist in dem nicht abschließend formulierten klarstellenden Benachteiligungsverbot auch eine Benachteiligung aus rassistischen Gründen erfasst; vgl. Gesetzentwurf der BReg., a.a.O., S. 20.
5) Der Entwurf geht an dieser Stelle zudem über den Tenor des Beschlusses des BVerfG hinaus.
6) Gesetzentwurf der BReg., a.a.O., S. 21.

Vor diesem Hintergrund stellt sich die Frage, wie bei Triage-Entscheidungen das Merkmal der Gebrechlichkeit auszulegen ist. An dieser Stelle lohnt ein Blick in das Betreuungsrecht (§ 1896 BGB) sowie das Verwaltungsverfahrensgesetz (§ 16 VwVfG). So hat das Betreuungsgericht einen Vertreter bzw. Betreuer zu bestellen, falls eine Person aufgrund einer psychischen Krankheit oder einer körperlichen, geistigen oder seelischen Behinderung ihre Angelegenheiten ganz oder teilweise nicht besorgen kann. In der Literatur wird in diesem Zusammenhang auch von *gebrechlichen* Beteiligten gesprochen[7]. Hierbei fallen unter körperliche Gebrechen etwa Lähmung, Blindheit, Taubheit, Bettlägerigkeit sowie allgemeine Altersgebrechlichkeit. Unter geistigen Gebrechen ist eine Minderung der geistigen Kräfte zu verstehen, wobei diese einen erheblichem Umfang haben müssen – so insbesondere bei Geisteskrankheit oder Geistesschwäche[8]. Die überwiegende Mehrzahl dieser Regelbeispiele dürfte auch die pönalisierten Merkmale des Alters sowie der Behinderung erfüllen. Es sind jedoch durchaus Konstellationen denkbar, bei denen eine auf intensivmedizinische Behandlung angewiesene Person gebrechlich ist, aber weder alt noch behindert ist. Insofern kommt dem Diskriminierungsmerkmal der Gebrechlichkeit eine eigenständige Bedeutung zu.

Schließlich ist die Aufzählung der in genannten Kriterien nicht abschließend („*insbesondere*"), sodass auch weitere Diskriminierungstatbestände in Betracht kommen können. Zwei weitere Kriterien, die einen Verstoß gegen das in § 5c Abs. 1 IfSG-E normierte Benachteiligungsverbot begründen können, sind z. B. die Berücksichtigung der Pflegebedürftigkeit oder aber der Sars-CoV-2-Impfstatus eines betroffenen Patienten[9]. Hierbei soll eine Rolle spielen, ob der Umstand, dass sich ein Patient gegen die Verabreichung einer Schutzimpfung entschieden und damit gegebenenfalls ein höheres Risiko für einen schwereren Verlauf einer übertragbaren Krankheit in Kauf genommen hat, verwirklicht hat. Im Hinblick auf den Impfstatus besteht zwischen der DIVI-Leitlinie sowie dem Triage-Gesetz Dissens: Hier wird der Impfstatus mit Verweis auf das Gleichheitsgebot als Priorisierungsgrundlage explizit ausgeschlossen[10].

2. Würdigung

Zunächst ist festzustellen, dass eine Bekräftigung des Diskriminierungsverbots von Art. 3 Abs. 3 S. 2 GG sowie § 1 AGG in § 5c Abs. 1 IfSG-E sinnvoll ist. Zudem geht die Fassung des § 5c Abs. 1 IfSG-E über einen reinen Programmsatz hinaus. So wird – wie ausgeführt – das Merkmal der Gebrechlichkeit eingeführt. Die Einführung dieses Benachteiligungsmerkmals ist grundsätzlich zu begrüßen[11]. Gleichwohl ist fraglich, inwiefern der Ausschluss des Merkmals der Gebrechlichkeit zur Feststellung der kurzfristigen Überlebenswahrscheinlichkeit praktisch anwendbar ist. So ist durchaus das Risiko gegeben, dass Gebrechlichkeit aus gewissen Komorbiditäten und Behinderungen geschlossen wird und die Ursachen der Gebrechlichkeit zwangsläufig zu einer schlechteren Einstufung der kurzfristigen Überlebenswahrscheinlichkeit führen[12]. Zwar ist bei der Bewertung der kurzfristigen Überlebenswahrscheinlichkeit nach § 5c Abs. 1 IfSG-E nicht mehr auf die Gebrechlichkeit als pönalisiertes Merkmal abzustellen, jedoch wären die Ursachen der Gebrechlichkeit weiterhin zur negativeren Bewertung der kurzfristigen Überlebenswahrscheinlichkeit zulässig. Dies setzt selbstredend voraus, dass Gebrechlichkeit und Überlebenswahrscheinlichkeit tatsächlich in einem direkten Zusammenhang stehen, was jedoch erfahrungsgemäß häufig der Fall sein wird. Im Ergebnis würde also die Gebrechlichkeit als Diskriminierungsmöglichkeit explizit ausgeschlossen, aber die Ursachen der Gebrechlichkeit bleiben bestehen und ihnen wohnt weiterhin ein erhebliches Diskriminierungspotenzial inne. Nach der Dogmatik des AGG würde es sich unter Umständen sogar um eine mittelbare Diskriminierung handeln, welche vorliegt, wenn dem Anschein nach neutrale Vorschriften, Kriterien oder Verfahren Personen wegen eines pönalisierten Merkmals gegenüber anderen Personen in besonderer Weise benachteiligen können.

In seiner Entscheidung hat das BVerfG festgestellt, dass *trotz* des eindeutigen Wortlauts des Art. 3 Abs. 3 S. 2 GG Menschen mit Behinderung bei der pandemiebedingten Triage einem Diskriminierungsrisiko unterlägen. Auch wurde vom Verfassungsgericht konstatiert, dass die S1-Leitlinie der DIVI bei pandemiebedingter Knappheit das Risiko einer Benachteiligung nicht beseitigen würden[13]. Dies gilt selbst, obwohl auch die S1-Leitlinie das Gleichheitsgebot aufgreift und eine Priorisierung aufgrund des Alters, Geschlechts, einer Behinderung, etc. ausschließt[14]. Zudem wurde bei einer von der Antidiskriminierungsstelle des Bundes in Auftrag gegebenen Untersuchung[15] festgestellt, dass Menschen mit Behinderung bei der Inanspruchnahme von medizinischen Leistungen Diskriminierungsrisiken unterlägen und das trotz des Diskriminierungsverbots nach Art. 3 Abs. 3 S. 2 GG und § 1 AGG. Demnach kann die Ergänzung des § 5c Abs. 1 IfSG-E allein nicht den Vorgaben des BVerfG gerecht werden, Diskriminierung von Menschen mit Behinderung bei pandemiebedingter Triage zu verhindern.

#### *II. § 5c Abs. 2 IfSG-E*

1. Inhalt

§ 5c Abs. 2 IfSG-E konkretisiert, wie eine Reduktion von Diskriminierungsrisiken erreicht werden soll. Hierfür werden Zuteilungskriterien definiert, respektive jene Kriterien benannt, welche die Zuteilungsentscheidung nicht beeinflussen dürfen. Die Zuteilungsentscheidung bei der Triage darf hiernach ausschließlich aufgrund der *aktuellen* und *kurzfristigen* Überlebenswahrscheinlichkeit der Patienten getroffen werden. Auf diese Weise werden zwar die grundsätzlichen Vorgaben der DIVI-Leitlinie[16], Zuteilungsentscheidungen an die Überlebenswahrscheinlichkeit, bzw. medizinische Erfolgsaussicht anzuknüpfen, übernommen, sie werden jedoch um das Kriterium der Aktualität sowie Kurzfristigkeit ergänzt. Maßgeblich für die Feststellung der aktuellen Überlebenswahrscheinlichkeit soll damit insbesondere der individuelle Gesundheitszustand des betroffenen Patienten im Zeitpunkt der Zuteilungsentscheidung sein[17]. Die aktuelle Überlebenswahrscheinlichkeit knüpft hierbei an die im Zeitpunkt der Zuteilungsentscheidung bestehende Aussicht an, die aktuelle Krankheit zu überleben. Durch das Abstellen auf die kurzfristige Überle-

7) Früher gab es zudem das Instrument der Gebrechlichkeitspflegschaft; hierzu: BGH, Beschl. v. 7. 11. 1984 – IVb ZB 830/81 –, NJW 1985, 433.
8) *Stelkens/Bonk/Sachs/Schmitz*, 9. Aufl. 2018, VwVfG § 16, Rdnr. 23.
9) Gesetzentwurf der BReg., a. a. O., S. 21.
10) *DIVI*, a. a. O., S. 5.
11) So auch *Gutmann/Fateh-Moghadam*, ZRP 2022, 130.
12) So zutreffend *Gutmann/Fateh-Moghadam*, ZRP 2022, 130, 131.
13) BVerfG, Beschl. v. 16. 12. 2021 – 1 BvR 1541/20 –, MedR 2022, 212.
14) *DIVI*, a. a. O., S. 5.
15) *Bartig/Kalkum/Le/Lewicki*, Diskriminierungsrisiken und Diskriminierungsschutz im Gesundheitswesen, https://www.antidiskriminierungsstelle.de/SharedDocs/downloads/DE/publikationen/Expertisen/diskrimrisiken_diskrimschutz_gesundheitswesen.pdf?__blob=publicationFile&v=2, 50, Zuletzt aufgerufen am 11. 2. 2023.
16) *DIVI* S1-Leitlinie AWMF-Registernummer 040-013, Entscheidungen über die Zuteilung intensivmedizinischer Ressourcen im Kontext der COVID-19-Pandemie, 3 überarbeitete Fassung v. 14. 12. 2021.
17) Gesetzentwurf der BReg., a. a. O., S. 21.

benswahrscheinlichkeit soll erreicht werden, dass eine Zuteilungsentscheidung nicht unter Berücksichtigung der mittel- oder langfristigen Lebenserwartung oder der Lebensqualität erfolgen darf[18].

Weitere Abweichungen zur DIVI-Leitlinie lassen sich bei denjenigen Kriterien feststellen, welche die Zuteilungsentscheidung nicht beeinflussen dürfen. Zwar sind Komorbiditäten weiterhin zur Beurteilung der (aktuellen und kurzfristigen) Überlebenswahrscheinlichkeit zulässig, jedoch nur, sofern sie diese *erheblich* verringern. Ausgeschlossene Kriterien bei der Entscheidungsfindung, welche sich nicht auf die Überlebenswahrscheinlichkeit auswirken, sind insbesondere Behinderung, Alter, verbleibende Lebenserwartung, Grad der Gebrechlichkeit und die Lebensqualität. An dieser Stelle bricht § 5c Abs. 2 IfSG-E teilweise mit der bislang gebräuchlichen DIVI-Leitlinie. Diese sieht vor, den Grad der Gebrechlichkeit (*Clinical Frailty Scale*, *CFS*) in die Entscheidung als Indikator für schlechtere Erfolgsaussichten miteinfließen zu lassen[19]. Auch sei anzumerken, dass die DIVI-Leitlinie bei der Entscheidungsfindung auf die Erfolgsaussicht der intensivmedizinischen Maßnahmen abstellt und gerade nicht auf die kurzfristige Überlebenswahrscheinlichkeit. Die Erfolgsaussicht ließe sich auch langfristig messen, was durch den eindeutigen Wortlaut des § 5c Abs. 3 IfSG-E ausgeschlossen wird. Außerdem schließt § 5c Abs. 2 IfSG-E explizit die sog. *ex-post-Triage* aus. Bereits zugeteilte Behandlungskapazitäten dürfen bei neuen Zuteilungsentscheidungen nicht miteinbezogen werden. Es dürfen also keine bereits zugeteilten Ressourcen den sich in intensivmedizinischer Behandlung befindenden Patienten entzogen werden, nur weil diese eine schlechtere kurzfristige Überlebenswahrscheinlichkeit aufweisen, als potenzielle Neuzugänge[20].

2. Würdigung

Vom BVerfG wurde befürchtet, dass das in den Leitlinien genannte Kriterium der klinischen Erfolgsaussicht im Sinne des Überlebens der aktuellen Erkrankung zu einem Einfallstor für eine Benachteiligung von Menschen mit Behinderungen werden könne. Das Triage-Gesetz geht auf diese Kritik insofern ein, als das nicht mehr auf die Erfolgsaussicht bei einer Zuteilungsentscheidung abgestellt werden darf, sondern auf die aktuelle und kurzfristige Überlebenswahrscheinlichkeit (vgl. § 5c Abs. 2 IfSG-E). Weiter wird konkretisiert, dass nur jene Komorbiditäten und Behinderungen berücksichtigt werden dürfen, welche die kurzfristige Überlebenswahrscheinlichkeit erheblich verringern. Fraglich ist, was unter einer erheblichen Verringerung verstanden werden kann. Bei einer *ceteris-Paribus*-Betrachtung von zwei Patienten, die sich ausschließlich dadurch unterscheiden, dass einer eine Behinderung aufweist, welche die kurzfristige Überlebenswahrscheinlichkeit leicht verringert, könnte geschlossen werden, dass nur durch diese Behinderung im Vergleich zu der Person ohne Behinderung die kurzfristige Überlebenswahrscheinlichkeit erheblich geringer ist, auch wenn sie dies ohne direkten Vergleich mit einer weiteren Person nicht der Fall wäre. Insofern ist anzuzweifeln, dass mit dieser gesetzgeberischen Ergänzung allein Diskriminierung von Menschen mit Behinderung – wie vom Verfassungsgericht *expressis verbis* gefordert – wirksam verhindert werden kann.

§ 5c Abs. 2 IfSG-E ist stark an die DIVI-Leitlinie angelehnt. Dennoch existieren einige wichtige Unterschiede zur DIVI-Leitlinie. Fraglich ist, ob diese Unterschiede zur Verhinderung der Diskriminierung von Menschen mit Behinderung ausreichen. Namentlich geht es um das verpflichtende Mehraugenprinzip sowie den verpflichtenden Konsens bei der Entscheidungsfindung (§ 5c Abs. 3 IfSG-E), der Vergleich kann jedoch zusätzlich auf Abs. 2 ausgeweitet werden, wo Kriterien, die keinen Einfluss auf die Entscheidungsfindung nehmen dürfen, sich teils mit für schlechte Erfolgsaussichten sprechende Kriterien nach der DIVI-Leitlinie decken[21]. Offensichtlich versucht der Gesetzgeber an dieser Stelle, die festgestellten Mängel der DIVI-Leitlinie zu heilen. Insbesondere das Hervorheben der Unzulässigkeit der Anwendung der *Clinical Frailty Scale* (vgl. § 5c Abs. 2, S. 3 IfSG-E) lässt diesen Rückschluss zu, da dieses Instrument auch im Beschluss des BVerfG ausdrücklich als diskriminierend dargestellt wurde, indem es nicht die besonderen Bedürfnisse von Menschen mit Behinderung adäquat berücksichtigt. Es wird mit der *Clinical Frailty Scale* insbesondere der Assistenzbedarf im Alltag gemessen, was bei Menschen mit Behinderung jedoch keine Rückschlüsse auf Gebrechlichkeit zulässt.

Zu Teilen kommt § 5c Abs. 2 IfSG-E den Forderungen des Sozialverbands Deutschland (SoVD) und des Deutschen Instituts für Menschenrechte (DIMR) nach, die im Rahmen ihrer Befragung als sachkundige Dritte[22] angaben, dass es einen Negativkatalog geben solle, der Kriterien erfasst, die bei einer Triage-Entscheidung keine Rolle spielen dürfen[23]. Zwar ist kein umfassender Katalog aufgestellt, jedoch schließt § 5c Abs. 2 IfSG-E die Berücksichtigung von Kriterien aus, die sich nicht auf die kurzfristige Überlebenswahrscheinlichkeit auswirken, wie insbesondere Behinderung, Alter, verbleibende Lebenserwartung, Grad der Gebrechlichkeit und Lebensqualität. Somit kann dies als kleiner Negativkatalog gewertet werden, woran Ärzte ihre Entscheidung ausrichten müssen. Es sei jedoch anzumerken, dass ein abschließender Negativkatalog auch nicht zielführend wäre. Ein solcher Katalog birgt ebenfalls ein Diskriminierungsrisiko, werden doch Personen, die keine im Negativkatalog aufgeführten Kriterien aufweisen, die knappen Ressourcen nicht zugewiesen, falls jemand mit Kriterien aus dem Negativkatalog mit ihnen um die Ressourcen konkurrieren sollte[24].

#### *III. § 5c Abs. 3 IfSG-E*

1. Inhalt

§ 5c Abs. 3 IfSG-E konkretisiert das personelle Vorgehen bei der Triage. Zuteilungsentscheidungen sind *einvernehmlich* von zwei mehrjährig intensivmedizinisch erfahrenen praktizierenden Fachärzten mit der Zusatzweiterbildung Intensivmedizin zu treffen. Zudem sind die Patienten unabhängig voneinander zu begutachten. Die S1-Leitlinie der DIVI empfiehlt zwar die Anwendung des Mehraugenprinzips, erwartet jedoch keine einvernehmliche Entscheidungsfindung. Sollte eine einvernehmliche Entscheidungsfindung nicht möglich sein, verlangt § 5c Abs. 3 IfSG-E die Miteinbeziehung eines weiteren, gleich qualifizierten Mediziners, sodass eine mehrheitliche Entscheidung getroffen werden kann. Die Beteiligung der weiteren gleichermaßen qualifizierten ärztlichen Person im Falle einer Meinungsverschiedenheit setzt keine körperliche Anwesenheit voraus. Eine telemedizinische Konsultation soll nach dem Willen des Gesetzgebers ausreichend sein[25]. So kann etwa

18) Gesetzentwurf der BReg., a. a. O., S. 21.
19) *DIVI*, a. a. O., S. 8.
20) Hierzu eingehend *Brettel/Schuhr*, MedR 2022, 801, 803; befürwortend zum Verbot der *ex-post*-Triage: *Gutmann/Fateh-Moghadam*, ZRP 2022, 130, 132.
21) *DIVI*, a. a. O., S. 8.
22) BVerfG, Beschl. v. 16. 12. 2021 – 1 BvR 1541/20 –, MedR 2022, 212, Rdnrn. 44, 46.
23) *Gelinsky*, https://www.kas.de/documents/252038/7995358/Was+regeln+in+einem+Triage-Gesetz+-+Zur+Zuteilung+von+%C3%9Cberlebenschancen+bei+unzureichenden+medizinischen+Ressourcen.pdf/c5e49a9e-f607-524e-d389-ae9b71fd9aee?version=1.0&t=1587487117224, zuletzt aufgerufen am 11. 2. 2023.
24) *Eufinger/Klemm*, GuP 2022, 161, 162.
25) Gesetzentwurf der BReg., a. a. O., S. 24.

ein qualifizierter Arzt eines anderen Klinikums, der telemedizinisch verfügbar ist, in die Entscheidungsfindung mit eingebunden werden.

Falls ein Patient mit Behinderung oder Komorbidität von der Zuteilungsentscheidung betroffen sein sollte, trifft § 5c Abs. 3 IfSG-E eine weitere Vorgabe. Zur Entscheidungsfindung muss eine Person hinzugezogen werden, die durch ihre Fachexpertise den besonderen Belangen des Patienten mit Behinderung oder Komorbidität gerecht werden kann. Auch diese Konsultation kann telemedizinisch stattfinden. Die fachkundige Person muss nach dem beredten Schweigen des Gesetzgebers kein Mediziner sein. Auch Pflegefachkräfte können hinzugezogen werden, da in der neuen Pflegeausbildung behinderungsspezifische Besonderheiten bereits zum Curriculum gehören.

2. Würdigung

Das geplante, verpflichtende Mehraugenprinzip nach § 5c Abs. 3 IfSG-E mit der damit verbundenen Pflicht zur einvernehmlichen Entscheidungsfindung ist positiv zu würdigen. Eine Entscheidung, wie die der Zuteilungsentscheidung, bei der es buchstäblich um Leben und Tod geht, sollte nicht von einem Arzt allein getroffen werden. Zwar ist es teilweise ohnehin bereits gängige Praxis, mit mehreren Ärzten die betroffenen Fälle zu besprechen und zu evaluieren, eine Verpflichtung zur gemeinsamen Entscheidung ist hier jedoch ein (weiterer) Schritt in die richtige Richtung. Insbesondere der Miteinbezug einer fachkundigen Person, sofern Menschen mit Behinderung oder Komorbiditäten von einer Zuteilungsentscheidung betroffen sein sollten, kann dem Anliegen, Diskriminierungsrisiken maßgeblich zu reduzieren, sollten sie auf fehlende Kenntnisse in diesem Bereich zurückzuführen sein, entsprechen. Nur so können die besonderen Bedürfnisse von Betroffenen hinreichend berücksichtigt und (unbewusste) Diskriminierung wegen fehlerhafter Stereotypisierung verhindert werden. In diesem Zusammenhang ist zudem positiv zu würdigen, dass der Gesetzgeber die Position von Pflegefachkräften stärkt, indem auch diese bei entsprechender Fachexpertise in die Entscheidung miteinbezogen werden können.

In der Gesetzesbegründung wird außerdem angekündigt, dass ergänzend die Approbationsordnung für Ärzte um „*Inhalte zu behinderungsspezifischen Besonderheiten*“ ergänzt werden soll. Hierfür soll das Gespräch mit der Bundesärztekammer gesucht werden, damit die Vorgehensweise in den Fällen der pandemiebedingt nicht ausreichenden Behandlungskapazitäten insbesondere in Fort- und Weiterbildung zeitnah vermittelt werden kann[26]. Dies ist ebenfalls ein wichtiger Schritt, um Diskriminierung von Menschen mit Behinderung wirksam und langfristig zu verhindern. So wurde bei einer Umfrage unter praktizierenden Medizinern im Februar 2022 festgestellt, dass 21,4 % der Befragten Fortbildungen als Regulierungsmöglichkeit der pandemiebedingten Triage am hilfreichsten empfinden, um auf diese Weise Stereotypisierung etc. vorzubeugen[27]. Wie vom Gesetzgeber intendiert, kann hiermit insbesondere das Risiko der unbewussten Stereotypisierung nachhaltig reduziert und mehr Fachwissen zu behinderungsspezifischen Besonderheiten aufgebaut werden[28]. Bei der neuen Ausbildungs- und Prüfungsverordnung für die Pflegeberufe ist dieses behinderungsspezifische Fachwissen bereits berücksichtigt[29]. Pflegefachkräfte erlernen in ihrer Ausbildung nicht nur, wie Menschen mit Behinderung fachgerecht unterstützt werden können, sondern auch, wie Kommunikationsbarrieren erkannt werden und welche kompensierenden Maßnahmen zur Überbrückung eingesetzt werden können.

#### *IV. § 5c Abs. 4 IfSG-E*

§ 5c Abs. 4 IfSG-E beschreibt die Vorgaben zur Dokumentation der Entscheidungsfindung und verweist auf § 630f BGB sowie § 630g BGB. Die neue Regelung erweitert eine bestehende Dokumentationspflicht lediglich um die Darstellung der maßgeblichen Umstände für die getroffene Entscheidung. Der Gesetzgeber geht davon aus, dass diese Dokumentation detailliert erfolgt, damit die behandelnde Person die Entscheidung nochmals reflektieren und die Zuteilungsentscheidung im Sinne der Transparenz nachvollzogen und geprüft werden kann[30]. Darüber hinausgehend soll sie den Beteiligten die Relevanz der Verfahrensvorschriften für eine diskriminierungsfreie sowie transparente Zuteilungsentscheidung verdeutlichen. In der Tat dient diese Vorgabe damit nicht nur den Interessen der von der Zuteilungsentscheidung betroffenen Patienten, sondern insbesondere auch den an der Entscheidungsfindung beteiligten Fachärzten[31]. Für die ärztliche Dokumentation wird vom Gesetzgeber ein Zeitaufwand von 30 Minuten angesetzt.

#### *V. § 5c Abs. 5 IfSG-E*

§ 5c Abs. 5 IfSG-E benennt schließlich die Verpflichtungen der involvierten Krankenhäuser. Diese sollen die ausgeführten Verpflichtungen in Verfahrensanweisungen sowie Verantwortlichkeiten festlegen. Außerdem stehen sie in der Pflicht, die organisatorische Umsetzung zu ermöglichen sowie die Einhaltungen der Verfahrensanweisungen sicherzustellen. Ferner sind die Verfahrensanweisungen jährlich zu prüfen und ggf. anzupassen. In diesem Zusammenhang wird erwartet, dass Ärzte auch die Aktualisierungen der Verfahrensanweisung hinreichend zur Kenntnis nehmen. Zudem müssen sich Ärzte, welche erstmalig in dem intensivmedizinischen Bereich eines Krankenhauses tätig werden, mit der jeweiligen Verfahrensanweisung vertraut machen. Die Vorschrift erlaubt es zudem jedem Krankenhaus, individuelle, eigene, auf lokale Gegebenheiten adaptierte Verfahrensanweisungen zu entwerfen und zu implementieren[32].

### C. Fazit

Auch wenn der Gesetzgeber betont, dass vorrangig alle Anstrengungen unternommen werden müssen, um den Fall pandemiebedingt nicht ausreichender intensivmedizinischer Behandlungskapazitäten zu verhindern[33], hat die Corona-Pandemie demonstriert, dass derartige Ausnahmesituationen niemals gänzlich auszuschließen sind. Für solche Situationen hat der Gesetzgeber nunmehr mit dem Triage-Gesetz ein Verfahren für die Zuteilung von überlebenswichtigen, pandemiebedingt nicht für alle verfügbaren intensivmedizinischen Behandlungskapazitäten geschaffen, welches Menschen mit Behinderung hinreichend berücksichtigt und ihr Diskriminierungsrisiko reduzieren soll. Das Triage-Gesetz enthält durchaus Regelungen, welche den Schutz einzelner Personengruppen vor Benachteiligungen bewirken können. Besonders positiv sind das Verbot der *ex-post*-Triage, das Mehraugenprinzip bei der Entscheidungsfindung sowie die explizite Nennung des Merkmals

26) Gesetzentwurf der BReg., a. a. O., S. 10.
27) *Eufinger/Klemm*, GuP 2022, 161, 163.
28) Gesetzentwurf der BReg., a. a. O., S. 10.
29) Vgl. PflAPrV, Anlage 2 bis 4, I.6.b und II.1.e.
30) Gesetzentwurf der BReg., a. a. O., S. 25.
31) Gesetzentwurf der BReg., a. a. O., S. 25.
32) Gesetzentwurf der BReg., a. a. O., S. 26.
33) Staatliche Gegenmaßnahmen waren etwa die Hospitalisierungsrate, die gesetzliche Pflicht, elektive Eingriffe und Behandlungen zu verschieben, um mehr Kapazitäten für an COVID-19 erkrankten Patienten zu haben sowie die Verteilung betroffener Patienten in andere Krankenhäuser nach dem zur Verlegung von COVID-19-Intensivpatienten konzipierten sog. „*Kleeblattkonzept*“.

der „Gebrechlichkeit" hervorzuheben. Entscheidendes Kriterium für eine Zuteilungsentscheidung darf nunmehr nur noch die aktuelle und kurzfristige Überlebenswahrscheinlichkeit des betroffenen Patienten sein. Gleichwohl ist nicht von der Hand zu weisen, dass auch durch das Kriterium der Überlebenswahrscheinlichkeit für bestimmte Personengruppen unvermeidbare Nachteile entstehen können, wohnt doch diesem Merkmal ein gewisses Benachteiligungspotenzial inne. So haben junge Menschen im Verhältnis zu alten und gebrechlichen Mensch sicherlich in der Vielzahl der Fälle eine höhere Wahrscheinlichkeit einer kurzfristigen Überlebenswahrscheinlichkeit. Unter Umständen handelt es sich beim Kriterium der aktuellen und kurzfristigen Überlebenswahrscheinlichkeit sogar um eine mittelbare Diskriminierung, welche jedoch wahrscheinlich sachlich gerechtfertigt werden kann. Ebenfalls ist am Triage-Gesetz zu kritisieren, dass bei Missachtung der Verfahrensregeln keine Sanktionen vorgesehen sind. Zudem stellt man sich die Frage, warum ausweislich des Gesetzeswortlauts nur die Triage bei Infektionskrankheiten geregelt wird, nicht aber für Fälle wie Naturkatastrophen oder Terroranschläge. Hier wird jedoch in einer entsprechenden Ausnahmesituation sicherlich eine analoge Anwendung in Betracht kommen.

**Open Access.** Dieser Artikel wird unter der Creative Commons Namensnennung 4.0 International Lizenz veröffentlicht, welche die Nutzung, Vervielfältigung, Bearbeitung, Verbreitung und Wiedergabe in jeglichem Medium und Format erlaubt, sofern Sie den/die ursprünglichen Autor(en) und die Quelle ordnungsgemäß nennen, einen Link zur Creative Commons Lizenz beifügen und angeben, ob Änderungen vorgenommen wurden.
Die in diesem Artikel enthaltenen Bilder und sonstiges Drittmaterial unterliegen ebenfalls der genannten Creative Commons Lizenz, sofern sich aus der Abbildungslegende nichts anderes ergibt. Sofern das betreffende Material nicht unter der genannten Creative Commons Lizenz steht und die betreffende Handlung nicht nach gesetzlichen Vorschriften erlaubt ist, ist für die oben aufgeführten Weiterverwendungen des Materials die Einwilligung des jeweiligen Rechteinhabers einzuholen.
Weitere Details zur Lizenz entnehmen Sie bitte der Lizenzinformation auf http://creativecommons.org/licenses/by/4.0/deed.de.

## Die Bereitstellung von Daten aus klinischen Prüfungen

### Auswirkungen der EU-Clinical Trials Verordnung und des EU-Data Governance Act auf Investigator Initiated Trials (IITs)

**Christoph Gerst, Sven Geyken und Raees Ahmed***

#### Abstract

In klinischen Studie wird eine Vielzahl an Daten und schutzrechtsfähigen Ergebnissen generiert. Diese können an sich einen wirtschaftlichen Wert besitzen. Vorliegend wird aus der Perspektive einer deutschen Hochschule, die eine (öffentlich geförderte) klinische Studie in eigener Verantwortung als sog. Investigator Initiated Trial (IIT) durchführt, betrachtet, welche dabei entstehenden Daten durch die Hochschule kommerzialisiert werden können. In diesem Zusammenhang legen die Autoren ein besonderes Augenmerk darauf, ob sich aus dem derzeitigen Datennutzungsgesetz und dem ab September 2023 geltenden Data Governance Act der EU (VO 2022/868) Einschränkungen für die Vergabe von exklusiven Lizenzen zur Nutzung dieser in der klinischen Studie generierten Daten ergeben.

Dr. phil. Christoph Gerst, M.A., Rechtsanwalt (Syndikusrechtsanwalt), Leitung Stabsstelle Wissenschaftsrecht,
Georg-August Universität Göttingen, Stiftung öffentlichen Rechts, Universitätsmedizin Göttingen, -Stabsstelle Wissenschaftsrecht-, v. Bar-Str. 2/4, 6. Stock, Raum 6.120,
37075 Göttingen, Deutschland

Sven Geyken, Referendar am OLG Braunschweig,
Universitätsmedizin Göttingen, -Stabsstelle Wissenschaftsrecht-, Göttingen, Deutschland

Dr. rer. medic. Raees Ahmed, LL.M., M.Sc., M.A., M.A., Dipl.-Ing. (FH)., Justiziar,
Sachgebietsleiter Forschungsrecht,
Universitätsmedizin Göttingen, -Stabsstelle Wissenschaftsrecht-, Göttingen, Deutschland

#### I. Einleitung

Bevor ein Medikament in den Verkehr gebracht, vulgo verkauft, werden kann muss es nachgewiesenermaßen sicher und wirksam sein. Beides wird anhand von wissenschaftlichen Experimenten untersucht. Werden solche Untersuchungen am Menschen mit dem Ziel durchgeführt *die klinischen, pharmakologischen oder sonstigen pharmakodynamischen Wirkungen eines oder mehrerer Arzneimittel zu erforschen oder zu bestätigen, jegliche Nebenwirkungen eines oder mehrerer Arzneimittel festzustellen oder die Absorption, die Verteilung, den Stoffwechsel oder die Ausscheidung eines oder mehrerer Arzneimittel zu untersuchen*[1], spricht man von einer „klinischen Studie" im Sinne der VO 536/2014[2].

Ein Sonderfall der klinischen Studie sind die sog. klinischen Prüfungen nach Art. 2 II Nr. 2 VO 536/2014, bei denen nicht nur zusätzliche Daten von den Prüfungsteilnehmern neben der üblichen Behandlung gesammelt werden, sondern wo schon die Behandlung selbst experimentell ist. Das bedeutet, dass das Medikament nicht für das entsprechende Krankheitsbild zugelassen ist, die Dosierung nicht zugelassen ist oder die Behandlung erfolgt nicht nach den jeweiligen ärztlichen Einschätzungen vor Ort, sondern statisch nach einem vorab festgelegten Prüfplan. Ziel einer

*) Die Autoren danken Herrn Rechtsanwalt *Marius Watermann*, Frau *Anna-Lena Hämmerich*, Frau *Louisa Jahn* und Herrn *Maximilian Rach* für die fachmännische und kritische Durchsicht sowie die anregenden Diskussionen.

1) Art. 2 II Nr. 1 VO 536/2014.
2) Verordnung (EU) Nr. 536/2014 des Europäischen Parlaments und des Rates vom 16. 4. 2014 über klinische Prüfungen mit Humanarzneimitteln und zur Aufhebung der Richtlinie 2001/20/EG, ABl. L 158 S. 1, ber. ABl. 2016 L 311 S. 25.

Funding

Open access funding enabled and organized by Projekt DEAL.

